# Increased long-term expression of pentraxin 3 in irradiated human arteries and veins compared to internal controls from free tissue transfers

**DOI:** 10.1186/1479-5876-11-223

**Published:** 2013-09-23

**Authors:** Tinna Christersdottir Björklund, Sarah-Jayne Reilly, Caroline Gahm, Barbara Bottazzi, Alberto Mantovani, Per Tornvall, Martin Halle

**Affiliations:** 1 Department of Medicine, Center for Molecular Medicine, Karolinska Institutet, Karolinska University Hospital, 171 76, Stockholm, Sweden; 2 Department of Oto-Rhino-Laryngology, Head and Neck Surgery and Department of Clinical Sciences, Intervention and Technology (CLINTEC) Karolinska Institutet, Karolinska University Hospital, 171 76, Stockholm, Sweden; 3 Department of Clinical Science and Education, Södersjukhuset, Sjukhusbacken 10, 118 83, Stockholm, Sweden; 4 Department of Molecular Medicine and Surgery, Section of Reconstructive Plastic Surgery, Karolinska Institutet, Karolinska University Hospital, 171 76, Stockholm, Sweden; 5 Humanitas Clinical & Research Center, via Manzoni 113, 20089, Rozzano, Milan, Italy; 6 Department of Translational Medicine, Università degli Studi di Milano, via Manzoni 113, 20089, Rozzano, Milan, Italy

**Keywords:** PTX3, CRP, Radiotherapy, Human, Blood vessels, Gene expression, Cardiovascular disease, Atherosclerosis, Stroke and neck

## Abstract

**Background:**

Clinical studies have shown that radiotherapy increases the risk of cardiovascular disease at irradiated sites years after exposure. However, there is a lack of biological explanations in humans. We therefore examined human blood vessels exposed to radiotherapy and studied C-reactive protein (CRP) and pentraxin 3 (PTX3), a new marker for adverse cardiovascular outcome dependent on TNF- alpha (TNFα) or interleukin-1beta (IL-1β) expression.

**Methods:**

Pairs of irradiated and non-irradiated human conduit arteries and veins were harvested from the same patient during autologous free tissue transfer for cancer-reconstruction at a median time of 48 weeks after radiotherapy. Differential gene expression was studied using qRT-PCR, confirmed by immunohistochemistry and cellular origins determined by immunofluorescence.

**Results:**

Gene expression in irradiated arteries compared to non-irradiated showed a consistent up-regulation of PTX3 in all patients and in a majority of veins (p < 0.001). Both TNFα and IL-1β were increased in irradiated compared to non-irradiated arteries (p < 0.01) and IL-1β correlated to the PTX3 expression (p = 0.017). Immunohistochemical and immunofluorescence staining confirmed an increased expression of PTX3 in endothelial cells, macrophages and smooth muscle cells.

**Conclusions:**

The sustained expression of PTX3 in arteries and veins tie biological evidence in humans to clinical studies and encourage further exploration of innate immunity in the pathogenesis of a radiation-induced vasculopathy.

## Background

Recent clinical studies show that radiotherapy against cancer increases the risk of cardiovascular diseases at the irradiated site, i.e. increased risk for stroke and acute myocardial infarction in patients treated with radiotherapy to the head/neck area and left thorax area, respectively [1-4]. This is further supported by animal experiments where radiotherapy accelerates the development of atherosclerotic lesions and induces an inflammatory plaque phenotype in ApoE−/− mice [5]. However, there is a paucity of studies in humans and animal experiments are limited by short life cycles since adverse clinical outcomes are not evident until years after radiotherapy exposure in the clinical situation [3,6]. As the incidence of cancer increases and cancer patients survive longer, further studies of human irradiated blood vessels are warranted to broaden the knowledge of these long-term complications of radiotherapy [7].

C-reactive protein (CRP), serum amyloid P component (SAP) and pentraxin 3 (PTX3) are all pattern recognition proteins belonging to the pentraxin super family, where CRP is a well established plasma marker for cardiovascular disease [8]. PTX3, on the other hand, is an interesting new plasma marker, because it is associated with cardiovascular disease severity, restenosis and has prognostic value in patients with ST-elevation myocardial infarction [9,10]. CRP and SAP are mainly produced in the liver in response to interleukin-6 (IL-6), however, CRP is also produced in small amounts in macrophages [11]. PTX3 is produced within the vessel wall in response to proinflammatory cytokines (especially interleukin-1beta (Il-1β) and tumor necrosis factor alpha (TNFα)) and Toll-like receptor (TLR) activation, but not IL-6 [12]. Several recent studies have shown that there is extensive local production of PTX3 in inflammatory vascular lesions, such as atherosclerotic plaques [13,14] and vasculitis [15]. Different cell types in the inflamed vascular wall, such as endothelial cells (ECs), smooth muscle cells (SMCs) and macrophages express PTX3 [14], but the function and the quantities of PTX3 expressed in different cell types is still unclear.

The aim of this study was to determine if previous radiotherapy of the neck is related to a sustained innate immune response that could explain recent epidemiological findings of ischemic stroke after neck irradiation [3]. During autologous free tissue transfer surgery, we have been able to harvest irradiated arterial branches from the carotid artery together with internal controls in humans. We aimed to study whether previous radiotherapy exposure is related to long-term increased gene expression of different pentraxins in the vessel wall. Furthermore, we wanted to study differences in inflammatory response between arteries and veins and identify cell types with an activated immune response after radiotherapy exposure, by measuring PTX3 expression.

## Methods

### Human blood vessel specimens

Irradiated and non-irradiated paired biopsies were harvested from the same patient during autologous micro-vascular free tissue transfer for reconstruction after cancer to the head neck area. The irradiated arteries and veins were harvested from side branches of the external carotid artery and the internal jugular vein respectively. The control vessels were harvested from the transferred tissue, i.e. radial femoral or fibular arteries and veins as indicated in Table 1. For gene expression studies, 20 pairs of arteries and 18 pairs of veins were examined. Demographic data of all patients, except for one arterial and one venous unidentified biopsy, are shown together with the radiotherapy treatment data (Table 1). Biopsies were harvested at a median time of 48 weeks (range 5–2100 weeks) after radiotherapy with a median dose of 64 Gy for both arteries and veins. None of the patients included in the study were treated with statins during the radiotherapy treatment. The paired conduit arteries and veins were freed from surrounding tissue under microscopic dissection peroperatively and stored in RNA-later® (Qiagen, Hilden, Germany) for gene expression studies, and formalin for immunofluorescence and immunohistochemistry studies. The study was approved by the Ethical Committee of Stockholm and was performed in agreement with institutional guidelines and the principles of the Declaration of Helsinki.

**Table 1:** Demographic and clinical characteristics

**Age**	XRT*	**Time after**	**Current smoking**	CVD***	**Biopsy**	**Control vessels**	**Fold change**	**Fold change**
**(yr)/sex**	**dose (Gy)**	XRT** (w)					**PTX3 A**	**PTX3 V**
60/M	64	500	no	0	A	forearm	5.8	-
77/F	54	5	yes	Past MI & CVL	A,V	forearm	28.6	39.2
47/F	68	7	no	0	A,V	forearm	323.1	2.7
68/M	64	48	no	Hypertension	A,V	forearm	29.8	0.2
60/M	50	6	no	Hypertension	A,V	fibula	7.7	36.2
63/M	68	9	no	0	A	fibula	5.1	-
64/M	64	2100	no	Hypertension	A,V	fibula	7.9	0.9
48/M	64	146	no	0	A,V	fibula	1.5	7.6
59/M	68	139	no	Past CVL	A,V	fibula	29.7	9.1
49/M	54	5	yes	0	A,V	forearm	12.4	15.4
50/F	60,3	22	no	Hypertension	A	forearm	66.5	-
50/M	64	90	no	0	A,V	forearm	120.3	55.2
59/M	54	7	yes	0	A,V	forearm	217.8	24.6
39/F	54	6	no	0	A,V	forearm	102.0	2.2
72/M	68	15	no	0	V	thigh	-	1.7
30/M	68	14	no	0	A,V	forearm	28.0	276.6
54/M	68	271	no	0	A,V	fibula	15.3	2.4
58/F	50	450	no	0	V	abdomen	-	52.8
73/F	64	170	no	0	A,V	fibula	63.0	46.3
62/M	68	290	no	Hypertension	A,V	fibula	15.7	29.8
63/F	66	650	yes	0	A	thigh	3.6	-

*Radiotherapy (XRT).

**Time from last XRT session to biopsy.

***Cardiovascular disease (CVD).

MI = myocardial infarction; CVL = cerebrovascular lesion.

A = Artery V = Vein.

### RNA extraction and cDNA synthesis

RNA was purified from human vessels using the RNeasy Mini kit® (Qiagen, Hilden, Germany) with on column DNase digestion step (Applied Biosystems™, Foster city, CA, USA). RNA concentration and quality were analyzed using NanoDrop 1000 Spectrophotometer (NanoDrop Technologies, Wilmington, DE, USA) and Agilent 2100 Bioanalyzer (Agilent Technologies, Inc., Santa Clara, CA, USA). cDNA synthesis was performed using total RNA with Super Script II reverse transcriptase® (Invitrogen™, Carlsbad, CA, USA).

### Gene expression

Gene expression method used was the real time polymerase chain reaction (qRT- PCR, TaqMan®, Applied Biosystems™, Foster city, CA, USA). The assays PTX3 (Hs00173615_m1, Applied Biosystems™, Foster city, CA, USA), CRP (Hs00357041_m1), SAP (Hs00356632_g1), TNFα (Hs00174128_m1), IL-10 (Hs00961622_m1), IL6 (Hs00985639_m1), TLR-4 (Hs001522939_m1) and IL-1β (Hs00174097_m1) were tested together with a standard curve to confirm the method reliability. The house keeping gene phosphoglycerate kinase 1 (PGK1) (Hs99999906_m1) was chosen based on results from a Taqman^®^ Endogenous Control Plate on three paired samples. Gene expression was calculated using the 2^-ΔΔct^ method [16] and PGK1 to compensate for intra and inter-qRT-PCR variations.

### Immunohistochemistry

Seven pairs of arteries and five pairs of veins were fixed in 10% formalin over night and embedded in paraffin. Paraffin embedded sections were cut and placed on super frost plus Menzel glasses (Thermo scientific, Wilmington, DE, USA). Dewaxing was performed in x-TRA-solv® (Medite® GmbH, Burgdorf, Germany) and rehydration in ethanol step wise.

Sections were pre-treated with DIVA dekloaker® 10× (Biocare Medical, Concord, CA, USA) for antigen retrieval. Selection and separation of tissue was made with 0.3% H_2_O_2_ (VWR International®, Radnor, PA, USA). Slides were blocked in 1:5 dilution of goat serum (DAKO, Carpintera, CA, USA) in PBS for 30 minutes at room temperature and then incubated with the primary antibody at 4°C over night. An affinity purified rabbit immunoglobulin G (IgG) against human PTX3 (1 mg/ml, 1:2000 dilution in phosphate-buffered saline (PBS), raised in Mantovani’s laboratory) [17] and a monoclonal mouse IgG against human CD68 (40 mg/l, 1:800 dilution in PBS, clone: PG-m1, DAKO, Carpintera, CA, USA) were used as primary antibodies.

Secondary antibodies used for CD68 and PTX3 were the polyclonal goat anti-mouse immunoglobulin/biotylated (DAKO, Carpintera, CA, USA) and the polyclonal goat anti-rabbit immunoglobulin/biotylated (DAKO, Carpintera, CA, USA), respectively. The secondary antibodies were added at a dilution of 1:5000 in PBS for 30 minutes in room temperature. The reactions were revealed using an avidin-biotin complex solution (ABC-solution, Vector laboratories Inc Burlingham, CA, USA) for 30 minutes at room temperature and 2 minutes with 3.3′-diaminobenzidine free base as a chromogen (DAB, Vector laboratories Inc Burlingham, CA, USA). Reactions were stopped with Millipore water. Hematoxcylin (Vector laboratories Inc Burlingham, CA, USA) was used to stain surrounding tissue. Dehydration was performed using x-TRA® solv and ethanol, step wise. Placenta was used as positive control for PTX3, tonsil for CD68 and PBS as a negative control. Microscope used for analysis was Nikon OPTIPHOT-2. Stained sections were analyzed pair wise in a blind manner by two individuals.

### Immunofluorescence

For double labelling, immunofluorescence studies were performed on eleven pairs of arteries. The tissue sections were deparaffinated by boiling in Citrate buffer (LabVision, Stockholm, Sweden) for 20 minutes and then cooled to room temperature for 20 minutes. After washing in PBS, sections were incubated with blocking serum (5% goat serum albumin in PBS) for 30 minutes at room temperature. Rabbit anti-PTX3 polyclonal antibodies (1:2000, Mantovani lab Humanitas, Milan, Italy), mouse anti-CD68 (1:800 DAKO, Carpintera, CA, USA), anti-alpha actin monoclonal mouse anti-human Smooth Muscle Actin (1:800, DAKO, Carpintera, CA, USA) and von Willerbrand factor (vWF) ( 1:400, DAKO, Carpintera, CA, USA) were used as primary antibodies. Negative controls were incubated in 1% Bovine serum albumin alone. Placenta, tonsil and arterial sections were used as positive controls for PTX3, CD68 and vWF/Alpha actin (α-actin), respectively. All sections were incubated with a mixture of the primary antibodies over night at 4°C. After washing with PBS, bound antibodies were visualized for PTX3 by use of a mixture of indocarbocyanine (Cy3)-conjugated goat anti-rabbit antibody (red colour, 1:1000, Jackson Immuno Research Laboratories Inc, Baltimore, PA, USA) and Alexa 488-conjugated donkey anti-mouse (green colour, 1:500, Molecular Probes, Stockholm, Sweden) applied for 60 minutes at room temperature. After washing with PBS, the nuclei of all sections were counterstained with 4′, 6-diamidino-2 phenylindole (DAPI) (blue colour, 1:10 000, Boehringer Ingelheim**,** Stockholm, Sweden) for 1 minute and sections were then mounted with glycerol: PBS (2:1). Evaluation of immunofluorescence staining was performed using a Leica DMRB™ fluorescence microscope with Leica filter cube L4.

### Statistics

Wilcoxon signed rank test was used to test differences between radiated and non-radiated arteries. Results with a p-value below 0.05 were considered to be statistically significant. Further correlations between PTX3 and IL-1β, TNF, IL-6, IL-10 and TLR-4 was analyzed with Pearson’s correlation.

## Results

**Figure 1:**
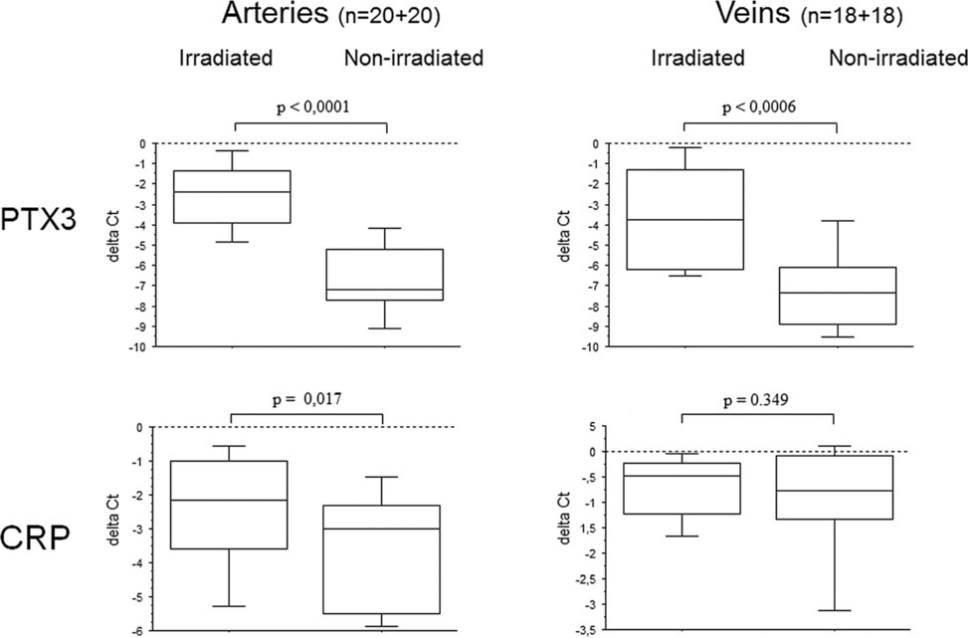
**Differential gene expression of PTX3 and CRP in arteries versus veins.** Gene expression of PTX3 and CRP in irradiated arteries and veins compared to non-irradiated internal controls, expressed as ΔCt-values (i.e. Ct-housekeeping gene - Ct-target gene).

**Figure 2:**
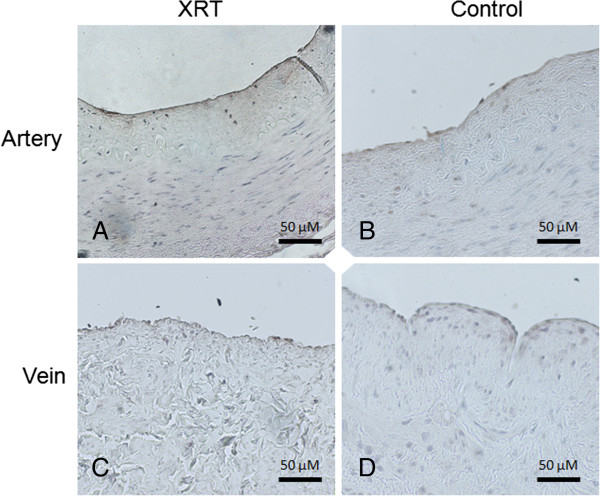
**PTX3 immunohistochemistry staining in paired human arteries and veins.** Immunohistochemistry with PTX3 staining in representative paired human arteries and veins from the same patient. The irradiated neck artery **(A)** shows positive staining, mainly confined to the endothelium compared to the non-irradiated radial artery **(B)**. A difference in PTX3 staining was less evident when an irradiated neck vein **(C)** was compared to the non-irradiated forearm vein from the same patient **(D)**. XRT = Irradiated vessel.

**Figure 3:**
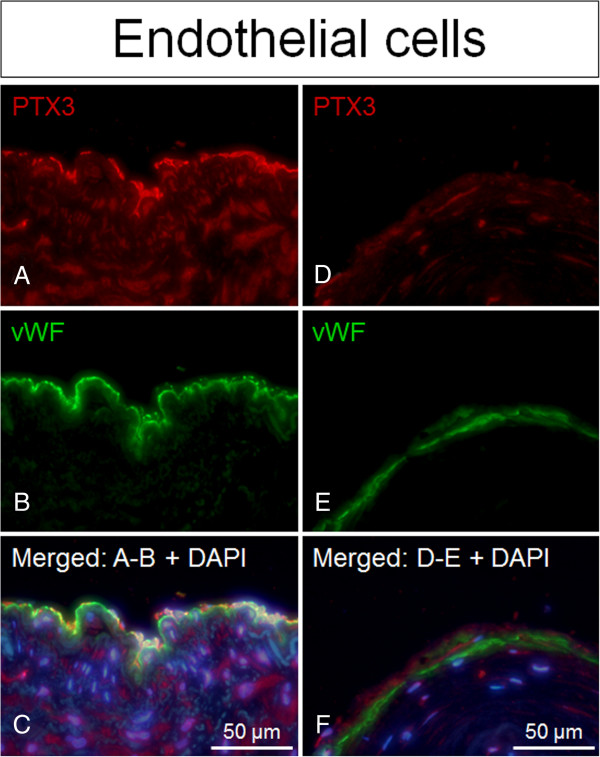
**PTX3 expression confined to ECs in irradiated versus non-irradiated arteries.** Positive staining of PTX3 (red fluorescence) superimposed with cell specific marker vWF (green fluorescence) indicated that PTX3 co-localizes with vWF/endothelial cells in irradiated arteries compared to non-irradiated arteries (controls), both from the same patient. Nuclear staining (blue fluorescence) was made with DAPI (C + F). **A**-**C** = irradiated vessels. **D**-**F** = non-irradiated vessel.

**Figure 4:**
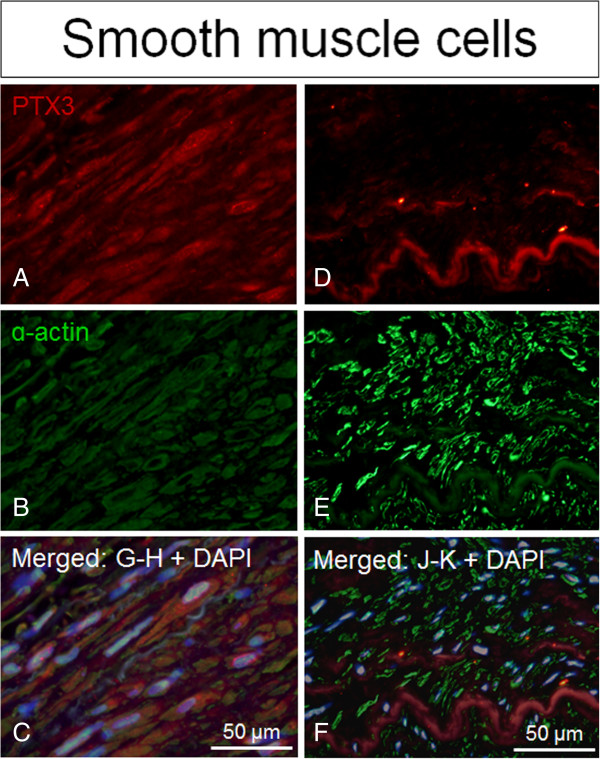
**PTX3 expression confined to SMCs in irradiated versus non-irradiated arteries.** Positive staining of PTX3 (red fluorescence) superimposed with cell specific marker α-actin (green fluorescence) indicated that PTX3 co-localizes with α-actin/smooth muscle cells in irradiated arteries compared to non-irradiated arteries (controls), both from the same patient. Nuclear staining (blue fluorescence) was made with DAPI (C + F). **A**-**C** = irradiated vessels. **D**-**F** = non-irradiated vessel.

**Figure 5:**
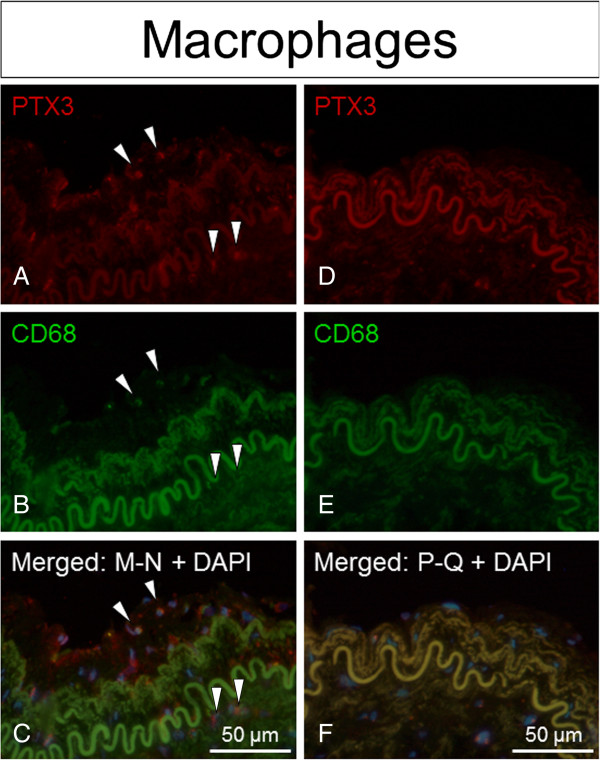
**PTX3 expression confined to macrophages in irradiated versus non-irradiated arteries.** Positive staining of PTX3 (red fluorescence) superimposed with cell specific marker CD68 (green fluorescence) indicated that PTX3 co-localizes with CD68/macrophages in irradiated arteries compared to non-irradiated arteries (controls), both from the same patient. Nuclear staining (blue fluorescence) was made with DAPI (C + F). **A**-**C** = irradiated vessels. **D**-**F** = non-irradiated vessel.

Table 1 shows the PTX3 fold change, demographic and clinical characteristics of the 21 enrolled patients (7 females, median age 60 years, and 14 males, median age 64 years). One arterial and one venous biopsy from an unidentified subject are not included in Table 1. PTX3 gene expression was increased in all 20 irradiated arteries (median 2^-ΔΔct^), was 4.39; Q1 2.74 – Q3 5.51; p < 0.0001) and in a majority of irradiated veins (median 2^-ΔΔct^ was 3.57; Q1 1.14 – Q3 5.29; p = 0.0006) compared to non-irradiated. Delta Ct values (ΔCt) for irradiated and non-irradiated vessels respectively are shown in separate box plots (Figure 1). Increased gene-expression of CRP was also observed to a smaller extent in irradiated arteries, but not in veins (Figure 1). There was no detectable gene expression of SAP (data not shown) and no significant relation between any increased gene expression and time from radiotherapy. Further, blinded analysis of immunohistochemistry results showed that PTX3 staining was stronger in all irradiated compared to non-irradiated, arteries and was mainly confined to ECs (Figure 2A-B). PTX3 staining was also seen in SMCs and macrophages, in irradiated arteries. However, PTX3 staining was only increased in some of the irradiated veins (Figure 2C-D). Positive immunofluorescence staining for PTX3 was observed to co-localize with staining for the cell type markers CD68 (macrophages), α-actin (smooth muscle cells) and vWF (endothelial cells), with the latter having the strongest co-localization signal observed (Figures 3, 4 and 5).

**Figure 6:**
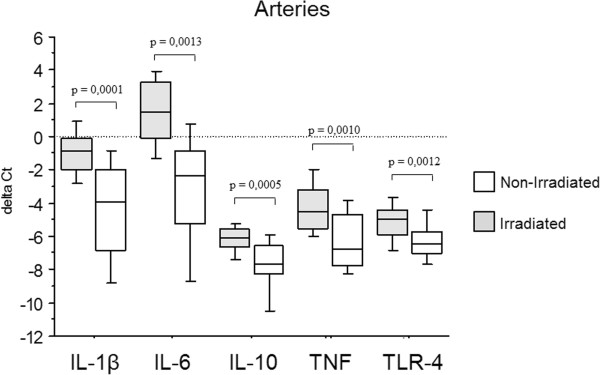
**Differential gene expression of IL-1β, IL-6, IL-10, TNF and TLR-4 in arteries.** Gene expression of IL-1β, IL-6, IL-10, TNF and TLR-4 in irradiated arteries compared to non-irradiated internal controls, expressed as ΔCt-values (i.e. Ct-housekeeping gene - Ct-target gene).

**Figure 7:**
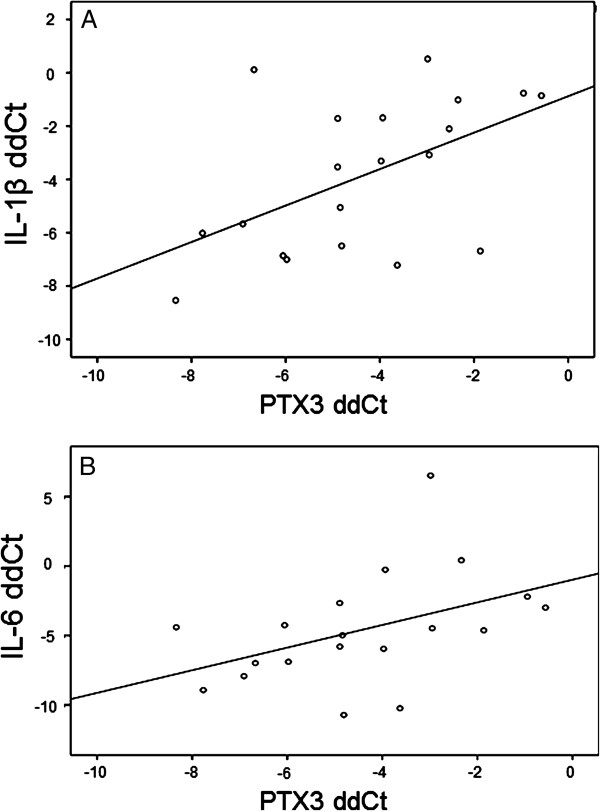
**Correlations between PTX3 and the target genes IL-1β and IL-6.** Correlations of genes expression data between PTX3 and IL-1β **(A)** and IL-6 **(B)** presented as ΔΔCt values (ΔCt irradiated – ΔCt non-irradiated).

A subanalysis of five additional target genes involved in innate immune response was performed in paired arterial biopsies. An increased gene expression was observed in irradiated, compared to non-irradiated, arteries for IL-1β (p = 0.0001), IL-6 (p = 0.0013), IL-10 (p = 0.0005), TNF (p = 0.0010) and TLR-4 (p = 0.0012) (Figure 6). A linear correlation was observed for 2^-ΔΔct^ -values between PTX3 and IL-1β (R = 0.53; p = 0.017) (Figure 7A) and trend between PTX3 and IL-6, (R = 0.44; p = 0.058), (Figure 7B).

## Discussion

In the present study we observed a consistently sustained upregulation of the novel cardiovascular disease marker PTX3 in all human irradiated conduit arteries and veins. By simultaneously harvesting paired biopsies from the same patient, we could exclude the influence of inter-individual differences and conclude that there was a significant increase of PTX3 expression due to irradiation only. This present study is important since it complements recent epidemiological studies showing that previous radiotherapy is a risk factor for cardiovascular disease, by providing biological evidence in humans. We suggest that the model is important for further investigations on long-term radiation-induced vasculopathy, emerging years after radiotherapy treatment in cancer survivors [2-4].

We have previously shown that irradiated arteries have sustained activation of nuclear factor-kappaB (NF-κB), a major mediator in innate immune response [18], but NF-κB is a broad transcription factor with multiple causes of action [19]. In order to determine if radiotherapy is a risk factor for cardiovascular disease, we chose to study PTX3. It is a new marker for vascular disease, and is associated with local activation of innate immunity and inflammation in atherosclerotic plaques [14,20] and cardiovascular disease severity [9]. Increased expression of PTX3 has previously been found in vascular inflammation, atherosclerosis and in the process of restenosis after angioplasty [10,13,20]. Expression of PTX3 is regulated by NF-κB, and therefore an appropriate down-stream target for further studies of radiation-induced vascular inflammation [20-22]. Results from this study support our hypothesis of a long-term increased arterial PTX3-related inflammatory response in ECs, SMCs and macrophages, similar to that seen in atherosclerotic plaques [14].

A link between radiotherapy and venous thrombosis has also been proposed, since radiotherapy leads to direct endothelial damage and the elicitation of an inflammatory response; both of which have been shown to be major mechanisms of coagulation activation. However, this link lacks clinical evidence, but is supported by a few clinical reports of axillary and subclavian vein thrombosis after radiotherapy [23] together with increased risk for graft failure due to venous thrombosis in microvascular anastomosis to irradiated vessels [4,24]. The present study shows that there is a sustained inflammatory activity also in veins, which may be linked to previous findings of endothelial dysfunction and prothrombotic properties in irradiated veins [18].

We also studied radiation-induced gene expression of CRP and SAP, two other pentraxins. The non-detectable levels of SAP were not surprising, since this molecule is not an acute phase protein in humans. However, the increased expression of CRP in irradiated arteries was rather surprising since CRP is usually not expressed within the vessel wall. Even though CRP expression was very low compared to PTX3, it suggests that there is local CRP-related inflammation at the irradiated site. CRP is mainly produced by hepatocytes and known for its systemic response to excessive vascular events, such as myocardial infarction. However, the small up-regulation seen in the irradiated arteries could be explained by the production of CRP from infiltrating macrophages in the inflamed arterial wall [8,14]. Our finding of infiltrating macrophages in irradiated arteries is supported both by our previous work [18] and by a study from Stewart and co-workers [25]. Together with the absence of infiltrating macrophages and CRP expression in veins, it is likely that macrophages are the source of CRP expression in irradiated arteries.

The more consistent and exaggerated increase in PTX3 expression in irradiated arteries compared to veins may in fact illustrate the difference in vessel morphology between arteries and veins. However, this may not only be explained by the ability of monocytes to infiltrate the arterial wall, but also by the higher content of SMCs in arteries compared to veins [25]. Immunohistochemistry staining revealed that increased PTX3 expression was confined mainly to ECs, but further immunofluorescence double staining of arteries showed PTX3 expression also in SMCs and macrophages. This is in accordance with the PTX3 expression seen in ECs, SMCs and macrophages in atherosclerotic plaques [14]. Taken together, the higher arterial SMC and macrophage content may explain our observed higher overall expression of both CRP and PTX3 in irradiated arteries compared to veins. There is a lack of studies comparing arterial to venous inflammation in humans. We therefore encourage further translational research with the described method in order to simultaneously compare the oxidative stress related immune response in respective vessel type.

Further analysis of IL-1β, IL-6, IL-10, TNF and TLR-4 showed an increased expression in irradiated compared to non-irradiated arteries for all genes, but the PTX3 expression was mainly correlated to IL-1β. It is known that both IL-1β and TNFα induce the PTX3 related innate immune response via NF-κB activation, but through different pathways. Our current data favours the hypothesis of an IL-1β mediated activation of innate immunity in this context, which is of interest in the future search for therapeutic adjuncts to cope with late adverse effects of radiotherapy, i.e. IL-1β receptor antagonists. The radiotherapy response with IL-1β associated PTX3 expression is furthermore supported by previous in vitro data [4,26]. This may partly be explained by the fact that irradiated tissues suffer from chronic oxidative stress with an increased production of reactive oxygen species (ROS) that further maintain a chronic IL-1β response [19,27]. A sustained deteriorated redox status with increased cell stress was in fact shown by us in a pathway analysis of global gene expression data comparing irradiated with non-irradiated arteries [18]. The up-regulation of IL-10 further support a chronic inflammatory process within the irradiated vessel wall [12].

The role of PTX3 in endothelial dysfunction is debated, where earlier studies have suggested PTX3 to have a detrimental role [28], whereas recent studies have also shown that PTX3 has an atheroprotective role [29] by inhibiting leukocyte adherence to the endothelium [21]. In this context, PTX3 was chosen to serve as biomarker for cardiovascular disease. However, whether there is a protective role of the observed consistent PTX3 up-regulation needs to be investigated in future studies.

The number of patients cured from cancer has increased over the past decades, and so has the interest in the long-term complications associated with cancer treatments. However, cardiovascular disease after radiotherapy is a relatively new entity with paucity of biological evidence in humans [2-4]. The results in this study clearly indicate that there is a sustained vascular inflammation in previously irradiated arteries and veins, even years after radiotherapy. Further knowledge about the pathology behind the long-term side effects of radiotherapy is necessary in order to develop methods to prevent the development of vascular damage at previously irradiated sites. We therefore believe that the present study will highlight the importance and indicate targets in the future search for therapy. The sustained PTX3 expression seen in irradiated arteries indicate that radiation-induced vascular inflammation shares similarities with atherosclerosis. Recent studies have shown the development of intimal hyperplasia in human arteries after radiotherapy [30], and since radiation-induced occlusions are comparable with occlusions developed by atherosclerosis [4], similar preventive treatment may be indicated. Systemic detection of PTX3 has been of prognostic value as observed in acute myocardial infarction [9], predicts adverse clinical outcome in patients with heart failure and is associated with coronary plaque vulnerability and furthermore decreased by statins [31]. However, the latter is still controversial since a recent study by Latini and co-workers describes increased PTX3 plasma levels in heart failure patients after rosuvastatin treatment [32]. Furthermore, in vasculitis PTX3 has been suggested a possible biomarker for disease activity [15]. Therefore further translational studies on plasma levels of PTX3 in the context of radiation-induced vasculopathy are needed.

Previous cell and animal experiments suggest that statins may be a future treatment for radiation-induced arterial inflammation [33]. In addition to the effect of decreasing levels of cholesterol in serum, statins also have anti-inflammatory properties, partly through inhibition of NF-κB activity. In recent in vitro studies, statins decreased PTX3 expression in ECs [34]. Gaugler and co-workers showed that Pravastatin limited endothelial activation after irradiation and decreased the inflammatory and thrombotic responses in ECs [33], but the effect on radiation-induced vasculopathy has yet not been tested in humans. Nonsteroidal anti-inflammatory drugs (NSAIDs) have been hypothesized as a possible treatment for radiation-induced vasculopathy, through its anti-inflammatory and anti-thromboembolic properties [35]. However, no cardioprotective effects have yet been shown for NSAIDs in the current context of radiation-induced vascular damage [36]. Animal experiments implicates that it is mechanistic differences between age-related and radiation-induced atherosclerosis and their response to NSAID treatment [36]. These findings encourage further studies on possible treatments against radiotherapy-induced vasculopathy.

Limitations of this study should be acknowledged. Gene expression analyses are made on full thickness vascular biopsies, reflecting the gene expression in several different cell types, i.e. ECs, SMCs and macrophages. However, further analysis with immunohistochemistry and immunofluorescence partly described which cell types are activated, but the exact contribution of PTX3 by each respective cell type has not been possible to study.

## Conclusions

To conclude, the present study shows that there is a sustained innate immune response with increased PTX3 expression in irradiated arteries due to a chronic activation of ECs, SMCs and macrophages within the vessel wall. The described method with analysis of carotid arterial branches with internal controls displays consistent results and represents one of a few biological studies in humans that strengthen recent epidemiological findings of stroke after neck-irradiation [3], and further show that radiotherapy is an independent risk factor for cardiovascular disease. With support from differential gene expression data with consistently regulated genes, we encourage further use of the described method in the future search for potential therapeutically targets against radiation-induced vasculopathy.

## Abbreviations

ROS: Reactive oxygen species; TNFalfa: Tumor necrosis factor alfa; NF-κB: Nuclear factor-kappaB; PTX3: Pentraxin 3; CRP: C-Reactive Protein; IL-1β: Interleukin-1beta; IL-6: Interleukin-6; IL-10: Interleukin-10; TLR: Toll like receptor; IHC: Immunohistochemistry; qRT-PCR: Real time polymerase chain reaction (Taqman®); PGK1: Phosphoglycerate kinase 1; PBS: Phosphate-Buffered saline; ABC-solution: Avidin-Biotin complex solution; DAB: 3,3′-diaminobenzidine; SMC: Smooth muscle cell; EC: Endothelial cell; NSAID: Nonsteroidal ant-inflammatory drugs.

## Competing interests

The authors declare that they have no competing interests.

## Authors’ contributions

TCB participated in the design of the study, carried out the immunohistochemistry and gene expression analyses, interpreted data, generated figures and drafted the manuscript. S-JR participated in the immunohistochemistry analysis and interpretation, and helped to draft the manuscript. CG carried out the immunofluorescence analysis and interpretation, and helped drafting the manuscript. BB and AM contributed by data interpretation, literature research and revision of the manuscript. PT participated in the design of the study, interpreted data, and helped with manuscript revision. MH conceived the study, and participated in its design and coordination, carried out the gene expression and statistical analysis, and performed data interpretation, contributed with literature research, generation of figures and writing of manuscript. All authors read and approved the final manuscript.
